# Machine learning‐directed electrical impedance tomography to predict metabolically vulnerable plaques

**DOI:** 10.1002/btm2.10616

**Published:** 2023-10-20

**Authors:** Justin Chen, Shaolei Wang, Kaidong Wang, Parinaz Abiri, Zi‐Yu Huang, Junyi Yin, Alejandro M. Jabalera, Brian Arianpour, Mehrdad Roustaei, Enbo Zhu, Peng Zhao, Susana Cavallero, Sandra Duarte‐Vogel, Elena Stark, Yuan Luo, Peyman Benharash, Yu‐Chong Tai, Qingyu Cui, Tzung K. Hsiai

**Affiliations:** ^1^ Department of Bioengineering, Henry Samueli School of Engineering University of California, Los Angeles Los Angeles California USA; ^2^ Division of Cardiology, Department of Medicine, David Geffen School of Medicine University of California, Los Angeles Los Angeles California USA; ^3^ Department of Medical Engineering California Institute of Technology Pasadena California USA; ^4^ Division of Cardiology, Department of Medicine Greater Los Angeles VA Healthcare System Los Angeles California USA; ^5^ Division of Laboratory Animal Medicine, David Geffen School of Medicine University of California, Los Angeles Los Angeles California USA; ^6^ Division of Anatomy, Department of Pathology and Laboratory Medicine, David Geffen School of Medicine University of California, Los Angeles Los Angeles California USA; ^7^ Division of Cardiothoracic Surgery, Department of Surgery, David Geffen School of Medicine University of California, Los Angeles Los Angeles California USA

**Keywords:** atherosclerosis, electrochemical impedance spectroscopy, machine learning, nanomaterials, oxidized low‐density lipoprotein

## Abstract

The characterization of atherosclerotic plaques to predict their vulnerability to rupture remains a diagnostic challenge. Despite existing imaging modalities, none have proven their abilities to identify metabolically active oxidized low‐density lipoprotein (oxLDL), a marker of plaque vulnerability. To this end, we developed a machine learning‐directed electrochemical impedance spectroscopy (EIS) platform to analyze oxLDL‐rich plaques, with immunohistology serving as the ground truth. We fabricated the EIS sensor by affixing a six‐point microelectrode configuration onto a silicone balloon catheter and electroplating the surface with platinum black (PtB) to improve the charge transfer efficiency at the electrochemical interface. To demonstrate clinical translation, we deployed the EIS sensor to the coronary arteries of an explanted human heart from a patient undergoing heart transplant and interrogated the atherosclerotic lesions to reconstruct the 3D EIS profiles of oxLDL‐rich atherosclerotic plaques in both right coronary and left descending coronary arteries. To establish effective generalization of our methods, we repeated the reconstruction and training process on the common carotid arteries of an unembalmed human cadaver specimen. Our findings indicated that our DenseNet model achieves the most reliable predictions for metabolically vulnerable plaque, yielding an accuracy of 92.59% after 100 epochs of training.

**Translational Impact Statement**

We developed a machine learning‐directed electrochemical impedance spectroscopy system and demonstrated its translational potential in the prevention of cardiovascular disease through its ability to assess the vulnerability of metabolically unstable plaques. The functionality and biocompatibility of our system was tested on ex vivo human models, such as an explanted heart. Accordingly, we were able to develop 3D reconstructions to describe the radial distributions of various atherosclerotic elements and implement convolutional neural networks to predict the likelihood of rupture. All our results were validated with histological data.

## 1 INTRODUCTION

Cardiovascular disease (CVD) remains one of the leading causes of death in developed countries, with over 19 million deaths reported globally each year.^1^ One of the root causes of CVD is atherosclerosis, or buildup of plaque, in the coronary arteries. Atherosclerotic lesions typically contain a necrotic core, a thin fibrous cap, macrophages, and calcification (Figure S1).^2^, ^3^ The necrotic core largely consists of metabolically active oxidized low‐density lipoprotein (oxLDL) crystals, whereas the fibrous cap consists of collagenous tissues that effectively serve as a protective layer to prevent the plaque from rupture. However, biomechanical forces, such as shear stress, and inflammatory factors, such as matrix metalloproteinase, can contribute to the thinning of the fibrous cap, leading to plaque destabilization and release of the lipid‐rich core into the bloodstream. Ultimately, this leakage can cause blood coagulation, or thrombosis, and hinder the flow of blood in the arteries.^4^

Detecting metabolically active and oxLDL‐laden plaques remains an unmet diagnostic challenge. Several imaging modalities, such as intravascular ultrasound and optical coherence tomography (OCT), have facilitated the characterization of atherosclerotic lesions, but these technologies are limited in their abilities to identify the metabolically active components of the atherosclerotic plaques, which harbor similar acoustic and scattering properties.^5^, ^6^ OCT also requires saline solution flushing to remove red blood cells in the aorta.^7^ Despite the ability to characterize oxLDL‐laden plaques, near‐infrared spectroscopy requires the injection of contrast agents.^8^ Despite high resolution to detect intraplaque hemorrhage and presence of lipid‐rich lesions, magnetic resonance imaging is bulky and costly.^9^ Multi‐slice spiral computed tomography allows for high‐resolution detection of calcification, but it exposes patients to radiation.^10^ To overcome these limitations when patients undergo elective angiograms, we have demonstrated both the theoretical and experimental bases of intravascular electrical impedance spectroscopy (EIS). This technique can reliably differentiate between the lipid‐rich core, fibrous cap, and calcification; thus, providing the sensitivity and specificity needed to characterize the vulnerability of the plaque.^11^ Intravascular EIS can be implemented by introducing an alternating current (AC) to the atherosclerotic lesion and measuring its impedance (Z) over a range of frequencies, where Z is a complex value consisting of resistance (R) as the real term and reactance (X) as the imaginary term (Z=R+jX). In addition, the amplitude of the AC current is typically below 10 mV, rendering this procedure safe and reliable.^12^

## 2 RESULTS AND DISCUSSION

### 2.1 Initial data from pig model

**FIGURE 1:**
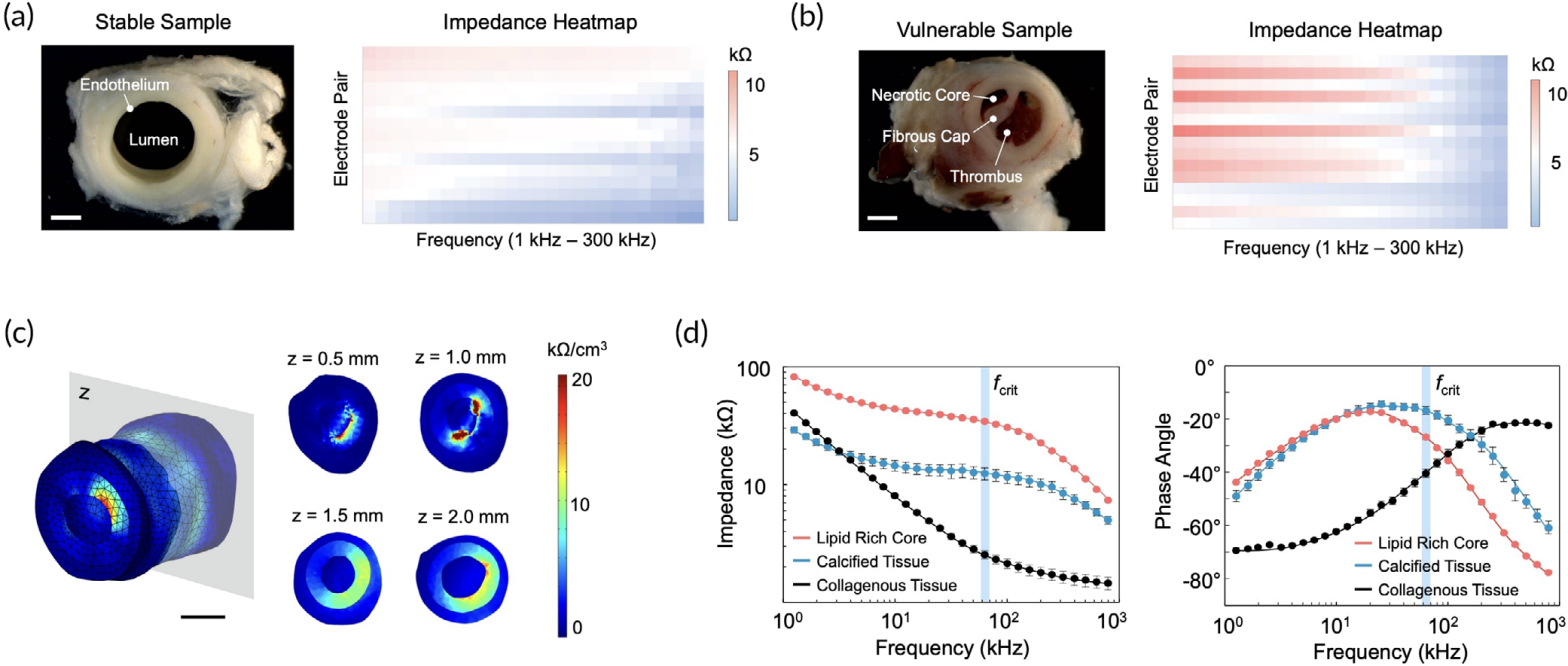
Initial data obtained from the RCCA of the Yucatan mini‐pig model. (a) The stable arterial sample consisted of healthy tissues, with no biomarkers of atherosclerosis. Its corresponding impedance heatmap suggests little obstruction to current flow. Scale bar: 1 mm. (b) Evidence of atherosclerosis, such as a necrotic core and fibrous cap, were present in the vulnerable sample. The presence of clotted blood, or thrombus, signifies a prior immune reaction. Moreover, the corresponding impedance heatmap suggests major obstructions to current flow. Scale bar: 1 mm. (c) A finite‐element model constructed from plaque‐laden histological segments shows the volume impedance density in 3D, representing the spatial distribution of oxLDL. Scale bar: 1 mm. (d) Averaged Bode plots (*n* = 6) of the three major atherosclerotic components confirm that they are best distinguished at the critical frequency (*f*_crit_) of 50 kHz. A greater weight was placed on impedance magnitude to simplify the training process of the machine learning models. However, phase data may also provide useful information regarding the electrical interactions between the atherosclerotic elements and the extracellular environment. oxLDL, oxidized low‐density lipoprotein; RCCA, right common carotid artery.

Initial results from the Yucatan mini‐pig model revealed several structural differences between the stable (Figure 1a) and vulnerable (Figure 1b) samples of the right common carotid artery (RCCA). For instance, the stable segment consisted of a clear lumen with healthy endothelial tissues, while the vulnerable segment contained biomarkers of oxLDL‐laden plaque, such as a necrotic core and fibrous cap. Clotted blood was also prevalent in the lumen of the vulnerable sample, suggesting that an immune reaction was induced by prior leakage of the necrotic core into the bloodstream. Accordingly, higher impedances were observed, suggesting that atherosclerotic components are more obstructive to current flow when compared to healthy tissues. Furthermore, finite‐element analysis of the oxLDL‐laden plaque in the RCCA segment revealed that the volume impedance density (VID) was most concentrated at the endothelial layer, with some influence at the collagenous tunica media (Figure 1c).

Upon measuring each atherosclerotic component of the pig model, we confirmed that the impedance of lipid‐rich cores, fibrous caps, and calcified tissues were best differentiated at 50 kHz. This value is a well‐accepted critical frequency for distinguishing biological tissues.^13^, ^14^ In our experiment, the lipid‐rich cores exhibited an impedance on the order of 30 kΩ, the calcified tissues on the order of 10 kΩ, and the collagenous tissues on the order of 2 kΩ (Figure 1d). Phase data also revealed noticeable differences at 50 kHz, with at least 10° of difference between each element.

### 2.2 Three‐dimensional EIT reconstruction

**FIGURE 2:**
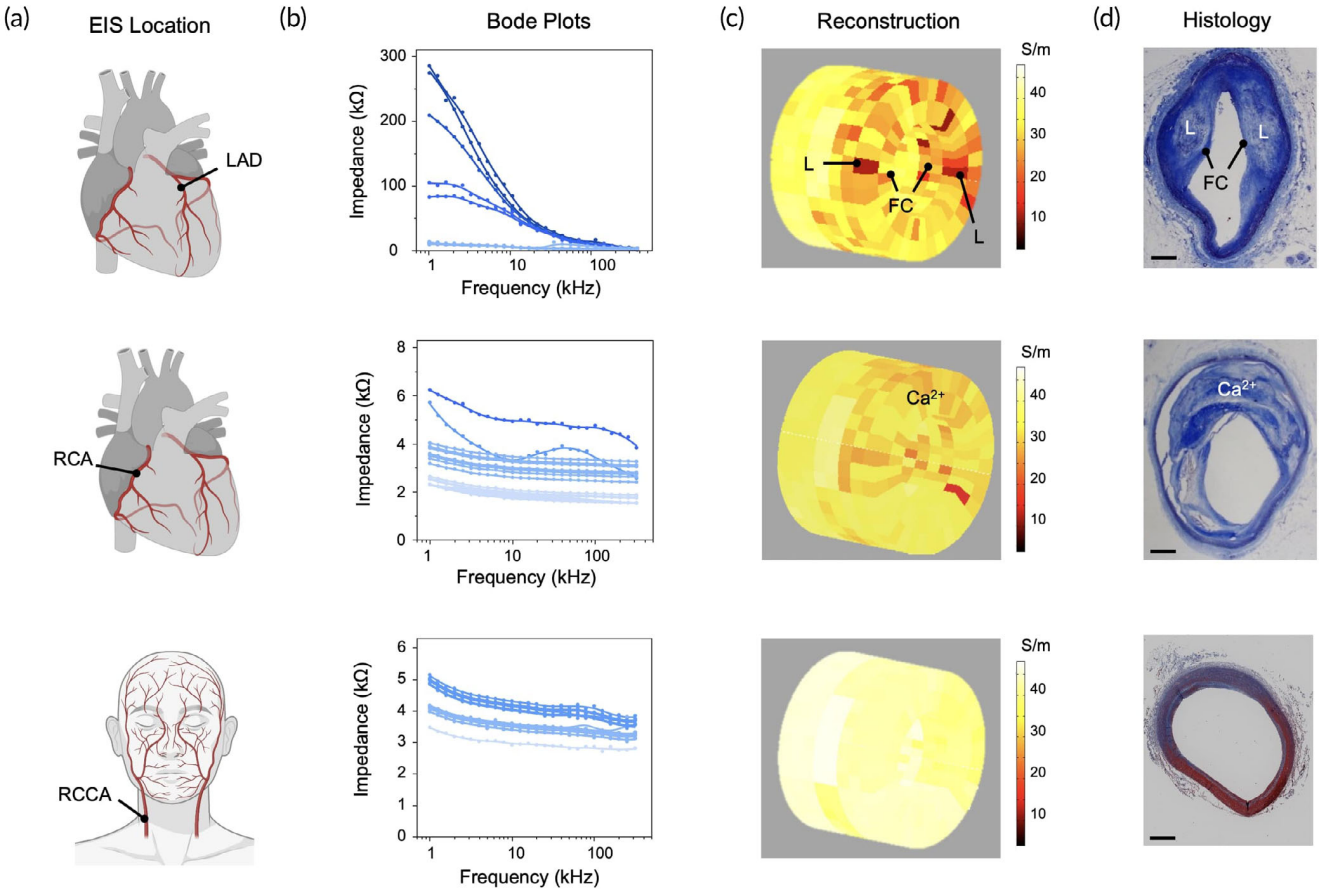
Three‐dimensional EIT reconstructions of EIS data. (a) EIS data were measured in the LAD and RCA of the explanted heart model, as well as the RCCA of the cadaver specimen. (b) Bode plots were acquired by iterating through the 15 combinations of electrode pairs, showing the impedimetric dependence on frequency. (c) Results from the well‐posed forward algorithm were used to render 3D conductivity maps, where red indicates regions of plaque buildup. Tomographic results predicted the presence of two lipid cores surrounded by fibrous caps in the LAD segment, prominent calcification in the RCA, and no signs of atherosclerosis in the RCCA. L: lipid core; FC: fibrous cap; Ca^2+^: calcification. (d) All histological results correlated with their corresponding EIT reconstructions, supporting intravascular EIS as a viable technique for plaque characterization. All scale bars: 1 mm. EIS, electrochemical impedance spectroscopy; EIT, electrical impedance tomography; LAD, left anterior descending coronary artery; RCA, right coronary artery; RCCA, right common carotid artery.

Combinatorial EIS data obtained from the left anterior descending coronary artery (LAD) and the right coronary artery (RCA) of the explanted human heart model, as well as the RCCA of the cadaver specimen (Figure 2a), served as the experimental data for our electrical impedance tomography (EIT) reconstruction. The corresponding Bode plots (Figure 2b) generally reported high impedances (>100 kΩ) from the LAD and low impedances (<10 kΩ) from both the RCA and RCCA. In accordance with initial results, 50 kHz was chosen as the critical frequency from which the conductivity values were derived. The 3D EIT reconstructions of the arterial segments were rendered using a red‐to‐yellow gradient colormap (Figure 2c), where red is indicative of regions with low electrical conductivity, corresponding to plaque buildup. Tomographic results suggested a high concentration of oxLDL‐rich plaque distributed between the upper‐left and upper‐right regions of the LAD and moderate calcification in the upper region of the RCA. Additionally, the RCCA of the cadaver specimen was found to be free of atherosclerosis. All EIT reconstructions aligned with measured EIS data and the corresponding immunohistology (Figure 2d).

### 2.3 Significance testing and data preprocessing

The results of our skewness test yielded a value of ${\tilde{\mu }}_{3}=0.8972$, with a greater number of stable lesions compared to vulnerable lesions in our data set. Accordingly, the difference of medians was chosen as the evaluation parameter for the null hypothesis significance test (NHST), which yielded a *p*‐value of <0.001 (Figure S2). Thus, we rejected our null hypothesis and determined that there existed a significant difference between the impedimetric measurements of vulnerable and stable plaques. Calculation of the 99% confidence interval (CI) showed that the difference in median impedances between these two categories likely fell between 10 kΩ and 17 kΩ. As an additional data preprocessing step, 3D principal component analysis (PCA) was able to cluster the impedimetric measurements based on underlying EIS features (Figure S3).

### 2.4 Evaluation of model performance

**FIGURE 3:**
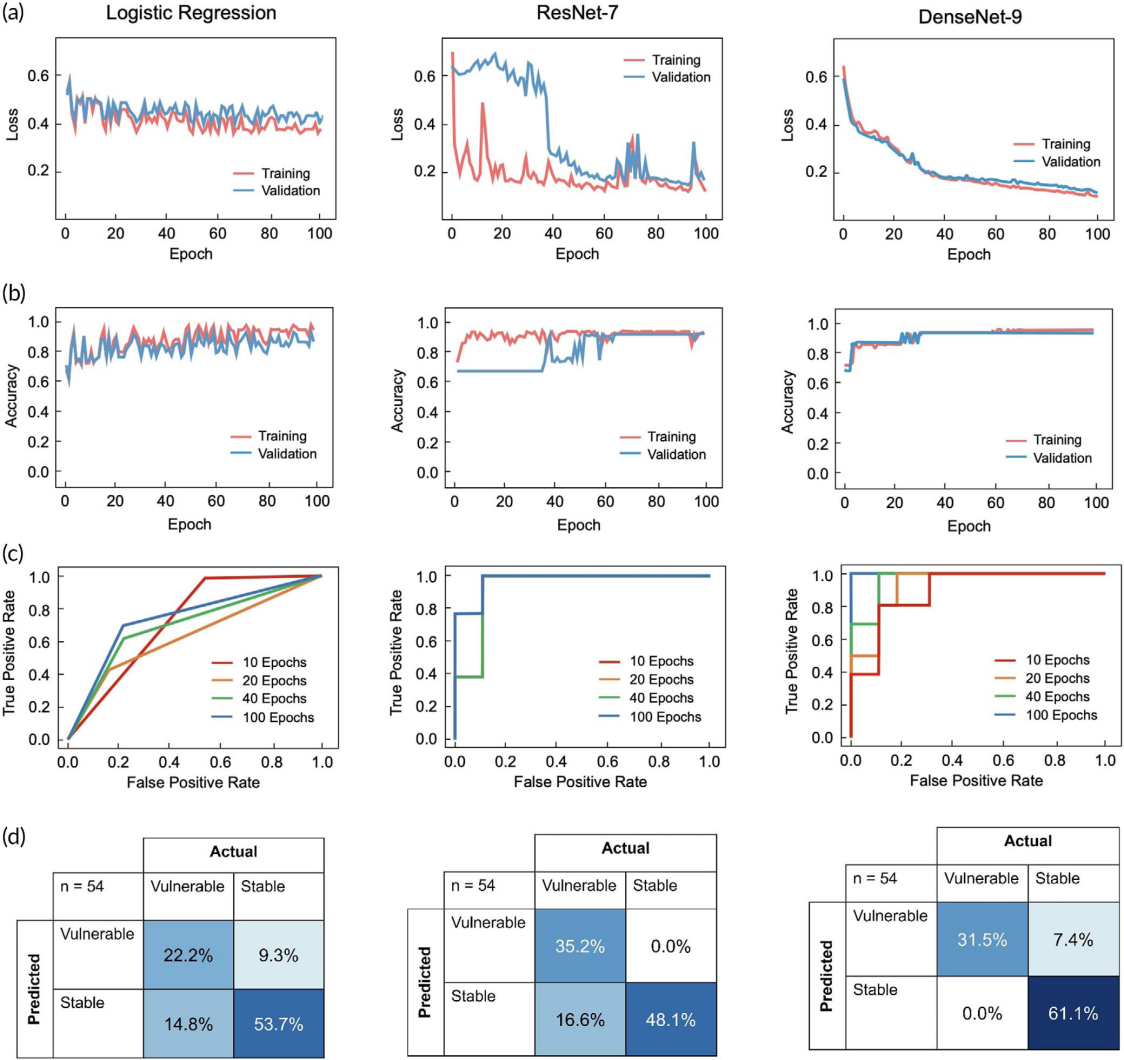
Performance metrics to evaluate the efficacy of logistic regression, ResNet‐7, and DenseNet‐9 in EIS classification. (a) Loss functions of all three models suggest that DenseNet‐9 yielded predictions with the most minimal error. ResNet‐7 also resulted in low error, but underfitting was experienced until 40 epochs of training. (b) Similarly, accuracy curves suggest that DenseNet‐9 yielded the most optimal predictions, with ResNet‐7 also converging after 40 epochs. (c) ROC curves were obtained over 10, 20, 40, and 100 epochs of training. One hundred epochs were sufficient to achieve AUCs of 0.75, 0.96, and 1.00 for the logistic regression, ResNet‐7, and DenseNet‐9 models, respectively. (d) Each prediction instance was categorized as a true positive, false positive, false negative, or true negative in the form of a confusion matrix. Percentages are rounded to the nearest tenth. AUC, area under the curve; EIS, electrochemical impedance spectroscopy; ROC, receiver operating characteristic.

Upon running the three models on a randomly generated testing and validation data set, DenseNet‐9 demonstrated the best performance, with a minimized loss function (Figure 3a) and a maximized accuracy curve (Figure 3b) when plotted against the number of epochs. ResNet‐7 initially demonstrated a discrepancy between the training and validation loss, which was indicative of underfitting, but these metrics began to converge at around 40 epochs. In contrast, the logistic regression model experienced fluctuations in both loss and accuracy, indicating that the model was not able to recognize enough patterns in the data set to achieve optimal results. A histogram comparing the validation results of all three models is shown in Figure S4. Next, receiver operating characteristic (ROC) curves revealed that 100 epochs of training resulted in the greatest amount of separability for each of the three models (Figure 3c). After running for this duration, DenseNet‐9 and ResNet‐7 achieved an area under the curve (AUC) of 1.00 and 0.96, respectively, signifying a low number of incorrect predictions within our validation data sets. The logistic regression model, on the other hand, produced a greater number of false positives, contributing an AUC of 0.75. Nonetheless, the AUC of all three models far exceeded that of a random classifier (AUC = 0.5), indicating their potentials in interpreting EIS data in the context of atherosclerotic vulnerability.

Further analysis of the confusion matrices (Figure 3d) suggested that DenseNet‐9 outperformed the other models in terms of accuracy, misclassification rate, sensitivity, and F_1_ score (Table 1), yielding 92.59% of correct classifications and no false negative predictions in the validation data set. ResNet‐7 also resulted in promising outcomes, yielding the most optimal specificity, false positive rate, and precision rates. In contrast, the logistic regression model generated inferior results across all metrics.

**TABLE 1:** Performance metrics for the evaluation of classification models.

	Logistic regression	ResNet‐7	DenseNet‐9
Accuracy	0.7593	0.8333	0.9259*
Misclassification rate	0.2407	0.1667	0.0741*
Sensitivity	0.6000	0.6786	1.0000*
Specificity	0.8529	1.0000*	0.8919
False positive rate	0.1471	0.0000*	0.1081
Precision	0.7059	1.0000*	0.8095
F_1_ score	0.6486	0.8085	0.8947*

*Note*: For each performance metric, the model with the desirable outcome is denoted with an asterisk (*).

Taken together, we determined DenseNet‐9 and ResNet‐7 to be the most appropriate models for evaluation of EIS data. DenseNet‐9 accumulated four incorrect predictions from our validation data set (*n* = 54), all attributed to false positives, while ResNet‐7 accrued nine incorrect predictions, which were all false negatives. However, the clinical context must be further investigated to determine whether DenseNet‐9 or ResNet‐7 is favorable. For instance, sensitivity is more desirable when performing EIS in essential arteries that supply blood to vital organs.^15^, ^16^, ^17^ Hence, DenseNet‐9 would be an appropriate model for such applications, as it maximizes sensitivity, which does not depend on the number of false positives. On the contrary, specificity is more desirable for noncritical arteries, especially in instances where secondary blood vessels are still able to deliver a sufficient blood supply to the target tissue.^16^ The number of false positive errors should be minimized, as they may lead to unnecessary medical interventions, posing a burden for the patient. Hence, ResNet‐7 would serve as the ideal model for this case, as it resulted in a lower number of false positive predictions compared to its counterparts.

### 2.5 Limitations and future directions

Despite the novel aspects of our machine learning‐guided EIS system, we acknowledge that there remains a need for continuous monitoring during interventional procedures, improving the signal‐to‐noise ratio (SNR), and clinical testing on patients. Currently, our reconstruction algorithm is limited to processing one set of data at a time, but future work could involve an iterative version of the reconstruction algorithm to continuously sample the tissue impedance and render a dynamic finite element model (FEM). To improve the SNR, we can investigate other nanomaterials, such as reduced graphene quantum dots (rGQDs), as a method of increasing the effective surface area at the electrode–tissue interface.^12^, ^18^, ^19^ Transmission electron microscopy performed in tangent to our initial animal experiments has verified the increased roughness of composite rGQD‐coated electrodes, resulting in an impedimetric reduction and a greater capacitance (Figure S5). Lastly, we must consider the biocompatibility of our system to ensure its successful translation to clinical use. Materials used in our EIS catheter, such as PtB, gold, polyimide (PI), and silicone, have all been found by previous studies to be safe for in vivo applications.^20^, ^21^, ^22^, ^23^ No adverse responses were observed in this study, and to the best of our knowledge, no immunogenic responses were reported in prior literature that tested similarly constructed EIS devices in vivo on animal models.^24^, ^25^, ^26^, ^27^ Despite the promising outlook of the materials implemented in our catheter, approval by the Food and Drug Administration (FDA) is still required to proceed with Phase I clinical study of our device.^28^

It is also important to note that only male Yucatan mini‐pigs were available during the time of experimentation. Thus, the data acquired during the initial portion of our study may not be entirely reflective of the female population. There are a few studies that suggest differences in atherosclerotic formation between males and females because of hormonal or dietary factors.^29^, ^30^ Hence, future studies should involve more rigorous testing of both sexes to fully examine the diagnostic capabilities of intravascular EIS sensors.

## 3 EXPERIMENTAL SECTION

### 3.1 Translational objectives

**FIGURE 4:**
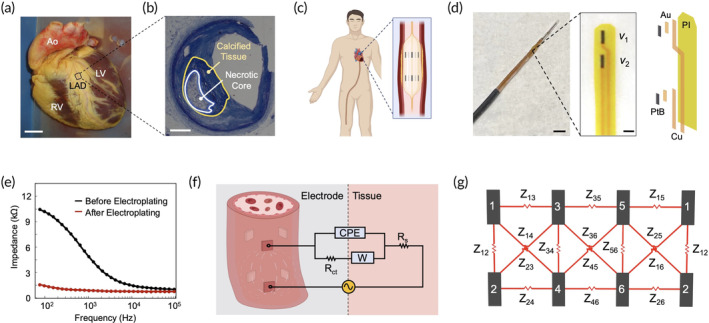
Experimental design and microelectrode configuration. (a) The explanted human heart used for this study. LAD: left anterior descending artery; Ao: aorta; LV: left ventricle; RV: right ventricle. Scale bar: 2 cm. (b) The corresponding immunohistology from the LAD reveals a lipid‐rich necrotic core surrounded by calcified tissue. Scale bar: 1 mm. (c) A six‐point microelectrode system was designed to be deployed from the femoral artery to the coronary arteries, which supply blood perfusion to the myocardium. (d) The gold (Au) microelectrodes were electroplated with platinum black (PtB) and deposited on a polyimide (PI) substrate. Copper (Cu) wires were embedded within the polyimide substrate to establish a direct connection to the electrochemical system. Here, v_1_ and v_2_ represent the recorded voltage signals. Scale bars (left to right): 1 cm, 1 mm. (e) An impedance spectrum illustrates a significant reduction in impedance at the low‐frequency regime after electroplating with platinum black. (f) The Randle's circuit serves as a well‐recognized model for electrophysiological stimulation, capturing the parasitic effects at the electrode–tissue interface. It is important to note that the electrodes are in contact with the endoluminal layer of the blood vessel wall. (g) The six‐point microelectrode configuration yields 15 different combinations of impedimetric measurements, which can be combined to form a 3D EIT reconstruction. EIT, electrical impedance tomography.

Previous EIS studies have largely been limited to the New Zealand white rabbit and mini‐pig models of atherosclerosis.^24^, ^25^, ^26^, ^27^ To demonstrate clinical translation in preparation for FDA‐regulated safety studies, we sought to interrogate the EIS profiles of the coronary arteries from the explanted hearts of patients undergoing heart transplant (Figures 4a and S6) and the carotid arteries from unembalmed human cadavers. We performed histological analyses as the ground truth (Figure 4b) to corroborate the 3D EIT profiles with the oxLDL‐laden plaques.

### 3.2 Electrode fabrication

In this study, a six‐point microelectrode‐based EIS sensor was developed around a silicone balloon catheter, with the eventual goal of delivery through the femoral artery (Figure 4c).^27^ The EIS sensor consisted of a 2‐by‐3 arrangement of gold (Au) electrodes that were electroplated with PtB, spaced 1.4 mm apart, and placed on top of a flexible PI substrate (Figure 4d). Copper (Cu) wires were also embedded within the PI substrate to establish a direct connection to the electrochemical workstation. By increasing the surface roughness of the electrodes at the nanoscopic level, electroplating serves to reduce the parasitic impedance at the low‐frequency regime (Figure 4e), which is primarily driven by the diffusion of ions at the electrode–tissue interface.^27^, ^31^ This phenomenon is known as the electrochemical double layer (Figure S7), and its impedimetric effects are modeled by Randle's equivalent circuit (Figure 4f). The nonlinear behavior of the interface is described by the constant phase element (CPE). Equation (1) represents its impedance, with α and $Y_{0}$ serving as tunable parameters.

(1) $$Z_{\mathrm{CPE}}=\frac{1}{Y_{0}{\left(\mathrm{j\omega}\right)}^{\alpha }},\;0\leq \alpha \leq 1,$$

A value of α=1 denotes an ideal capacitor with capacitance $Y_{0}$, while a value of α=0 denotes an ideal resistor with resistance $1/Y_{0}$.^32^ The Warburg diffusion element (*W*) is a specific case of the CPE where α=0.5. The impedance of the Warburg element can be derived from Fick's second law of diffusion, capturing the passive transport of ions through the electrochemical double‐layer.^33^

From the six‐point configuration, 15 different combinations of impedimetric measurements (Figure 4g) can be obtained for each arterial segment, allowing for the subsequent 3D EIT reconstruction. While the number of microelectrodes can be increased for future EIS designs, six points were chosen for this study because the proximity of the microelectrodes to each other would lead to an inherent tradeoff between EIT resolution and parasitic capacitance.^34^

### 3.3 Initial testing on the mini‐pig model of carotid atherosclerosis

To demonstrate the theoretical and experimental bases of EIS prior to human studies, we conducted a series of initial experiments using segments of the right carotid artery isolated from male Yucatan mini‐pigs (*n* = 6). Surgical ligation was performed, followed by feeding with a high‐fat diet for 16 weeks to develop atherosclerotic lesions in compliance with the protocol approved by the UCLA Institutional Animal Care and Use Committee (IACUC) #2014‐059. Statistical significance for this sample size was shown by a previous study, which yielded a *p*‐value of less than 0.05.^27^ As a proof‐of‐concept experiment, EIS data was acquired using a 10‐mV amplitude signal and swept across a frequency range of 1 kHz to 300 kHz at 10 points per decade. It was expected that the metabolically active and vulnerable atherosclerotic plaques are oxLDL‐rich and that these plaques would yield higher impedances throughout this range.

Next, a computational model was developed to visualize how impedance is distributed among the three tunics of the artery. Methods from Abiri et al. (2022) were implemented to create a three‐dimensional reconstruction (Figure S8) based on a set of 11 histological cross sections from an oxLDL plaque laden RCCA, each spaced 0.4 mm apart. The corresponding model was imported onto the AC/DC module of COMSOL Multiphysics, where each layer was assigned a conductivity (σ) and relative permittivity (ε) (Table S1). Then, a pair of electrodes were made in contact with the endoluminal wall, conforming to the same geometry as the EIS catheter, and activated with a peak‐to‐peak AC voltage of 25 mV across a frequency sweep of 1 kHz to 300 kHz. The VID was evaluated using Equation (2), where $J_{1}$ and $J_{2}$ denotes the current densities measured at the electrodes, ρ represents the resistivity, and I denotes the injected current.^34^

(2) $$\mathrm{VID}=\rho \frac{J_{1}\cdot J_{2}}{I^{2}}.$$

The VID was mapped onto the 3D model using a finite‐element solver. Four x‐y cross sections were obtained from the geometry to enhance the visualization of the interior impedimetric distribution, each spaced 0.5 mm apart with respect to the z‐axis.

### 3.4 Differentiation of atherosclerotic components

To demonstrate the 3D EIT data for the oxLDL‐laden endoluminal wall, we calibrated a frequency at which maximum differentiation was established between the main components of an atherosclerotic plaque. We isolated the three main components: namely, lipid‐rich cores, fibrous caps, and calcified sections of the arterial wall from the carotid arteries of the mini‐pig model of atherosclerosis (*n* = 6). EIS measurements of the endoluminal wall were obtained within a frequency sweep from 1 kHz to 1000 kHz, a wide experimental range to interrogate the characteristics of the impedance spectra of the three main components. A critical frequency was identified such that the resulting impedance magnitudes (Z) and phases (θ) of the lipid‐rich cores, fibrous caps, and calcified tissues were most distinguishable. However, to simplify the training process, a greater weight was placed on differentiating the magnitudes.

### 3.5 Reconstruction of the EIT

**FIGURE 5:**
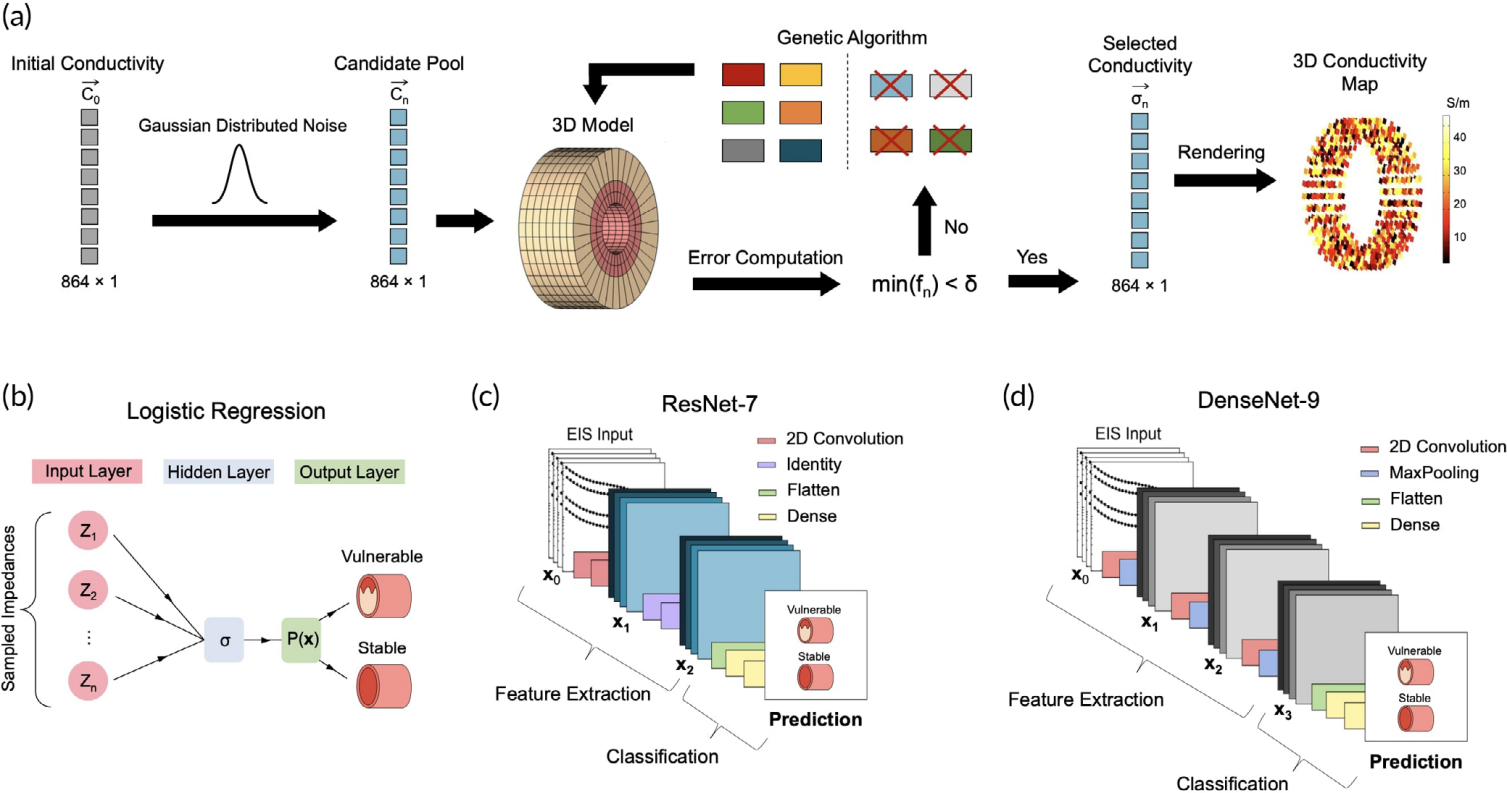
Architectures of algorithms and classification models for the analysis of EIS data. (a) The 3D EIT reconstruction algorithm consists of iterating the candidate pool through a genetic algorithm until its error function converges to a value below a predetermined threshold. Once this condition is reached, a separate program processes the final conductivity matrix and renders the corresponding model in a single step. (b–d) Model architectures of logistic regression, ResNet‐7, and DenseNet‐9, respectively. These models were evaluated against each other to determine the best architecture for EIS plaque classification. EIS, electrochemical impedance spectroscopy; EIT, electrical impedance tomography.

3D EIT was performed on 18 arterial segments of two ex vivo human models: an explanted heart and a cadaver. Vessels containing significant atherosclerotic buildup or stenosis were grounds for inclusion, while poorly preserved samples were excluded. Eight of the segments were acquired from the RCA and LAD of the explanted heart, while 10 of the segments were taken from the RCCA of the cadaver specimen. This study was approved by UCLA Institutional Review Board #17‐001112, with patient consent acquired prior to the research. To visualize the distribution of plaque within these human samples, we designed a cylindrical FEM, consisting of 3 coaxial layers, 8 rows, and 36 elements per row for a total of 864 elements. The coaxial layers represent the tunica adventitia, tunica media, and the plaque laden tunica intima. Unlike traditional EIT solvers that utilize the ill‐posed inverse problem, we implemented a well‐posed forward algorithm (Figure 5a) based on EIDORS (version 3.11) to solve for the conductivity matrix corresponding to each of the 864 elements, thereby enabling the 3D rendering of a plaque distribution map.^27^ The algorithm begins with an initial guess of the conductivity matrix, which was obtained by evaluating each of the 15 impedance functions at the critical frequency and by solving for the equations in Methods S1. Then, Gaussian‐distributed noise was added to the initial guess, thereby yielding the first set of candidates for the conductivity map $C_{n}$. We defined a fitness function f, as shown in Equation (3), and iterated through a “genetic algorithm” until its minima no longer exceeded our predefined error threshold δ.

(3) $$f=\sqrt{\sum_{i,j=1,i\neq j}^{15}{\left(Z_{\mathrm{ij},\text{measured}}-Z_{\mathrm{ij},\mathrm{sim}}\right)}^{2}}.$$

Once this condition was met, the values of the final conductivity matrix $\sigma_{n}$ were chosen as the basis for the 3D EIT reconstruction. To automate the process, we developed a MATLAB program that processes the conductivity matrix from a spreadsheet file and renders the corresponding FEM in a single step.

### 3.6 Establishment of the ground truth and data preprocessing

After obtaining the EIT reconstruction of the intravascular space, three classification models (logistic regression, ResNet‐7, and DenseNet‐9) were designed to predict the vulnerability of an atherosclerotic lesion based on EIS data. These models were chosen for their simplicity of training and predicting, as well as their incorporation of neural networks.^35^, ^36^ Logistic regression statistically evaluates the relationship between dependent and independent variables, rendering it promising for multi‐frequency EIS applications.^37^, ^38^ It often serves as a baseline to validate more advanced models.^39^ ResNet and DenseNet are widely regarded for image classification problems, and we speculated that they can also be generalized to predict plaque vulnerability from EIS data.^40^

Histological information was regarded as the ground truth for all three models. Previous staining methods were applied to the arterial segments to distinguish between oxLDLs, smooth muscle cells, and calcified tissues.^11^ A parameter known as the histological plaque vulnerability index (HPVI) was used to classify each cross section, as it is based on the descriptive classifications set forth by the American Heart Association (Table S2).^41^ An HPVI was internally assigned to each of the human samples in the model by comparing them to the published examples.^42^ Considering the binary nature of classification models, atherosclerotic lesions with a vulnerability index of ≥2 were regarded as vulnerable, while samples with a vulnerability index of <2 were regarded as stable.

Next, an NHST was conducted for all EIS spectra (*n* = 270) to ensure that different conditions of plaque were properly distinguished. For this study, the null hypothesis ($H_{0}$) proposes that no significant differences exist between the impedimetric measurements of vulnerable and stable plaques. The significance threshold was set at α=0.01, and the skewness $\left({\tilde{\mu }}_{3}\right)$ of the data set was evaluated to determine the type of distribution (Methods S2). The difference in means would be a more appropriate parameter for the evaluation of a normally distributed data set, while the difference in medians would be more appropriate for a skewed data set, as there exists more outliers. After performing the NHST, a 99% CI was constructed to demonstrate a likely range of values for which the chosen independent variable is to be found.

The initial data structure was multivariate, consisting of impedimetric measurements sampled from seven different frequencies in the 1 kHz–300 kHz range and the ground truth. Due to the complexity of the raw data, PCA was performed to reduce its dimensionality, while conserving as much variability as possible.^43^ This data preprocessing strategy reduces the need for computationally expensive processes by weighing only the most important features of the EIS spectra.^44^

### 3.7 Architectures of tested models

The architectures of three different classification models were modified to predict the vulnerability of atherosclerotic lesions based on the corresponding EIS data. The first model was a logistic regression classifier (Figure 5b), a widely used statistical technique that relies on the probability function $P\left(x\right)$ shown in Equation (4), where x refers to the sampled impedances and β refers to coefficients that can be optimized to yield the best fit.^45^ A threshold value $P_{\mathrm{th}}$ was predefined at 0.5. The model was designed to classify an atherosclerotic segment as vulnerable if the prediction yielded a value greater than $P_{\mathrm{th}}$ and as stable if the predicted probability was less than $P_{\mathrm{th}}$.

(4) $$P\left(x\right)=\frac{\exp\left(\beta_{0}+\beta_{1}x\right)}{1+\exp\left(\beta_{0}+\beta_{1}x\right)}.$$

However, one potential limitation of logistic regression is that it assumes a uniform relationship between the impedimetric data and the prediction outcome, which is often not the case with multi‐frequency EIS.^46^ To account for this, we evaluated the EIS data on ResNet‐7 (Figure 5c), a seven‐layer convolutional neural network that utilizes shortcut connections.^47^ Shortcut connections are a unique feature in which the outputs of one layer directly serve as the inputs of another layer. Moreover, seven layers were chosen after an optimization process on pseudo‐data. The first four layers were designed to perform feature extraction, consisting of two convolution layers, followed by a pair of identity layers. The convolution layers possess a set of 3 × 3 filters that act on the input EIS spectra, capturing important information, such as the critical and inflection points that are characteristic of a particular atherosclerotic component. Then, the identity layers relay the convolutional output to the next layer, known as the flattened layer. The flattened layer transforms this data into a one‐dimensional vector, which feeds into a pair of dense layers, where a set of weights are applied to perform classification.

To further add to our pool of classification models, we implemented DenseNet‐9 (Figure 5d), a nine‐layer neural network based on the original design of ResNet.^48^ The same optimization process was used to figure out the number of layers. However, the unique feature about DenseNet is that each of its layers receives inputs from all previous layers and passes its own outputs to all subsequent layers. Thus, it forms a dense network consisting of 45 different connections in our DenseNet‐9 model. The initial phases were designed to perform feature extraction, consisting of three pairs of convolution‐identity layers, arranged in an alternating manner. Then, the entire set of outputs was fed into the classification module, which also contained a flattened layer, followed by a pair of dense layers.

### 3.8 Metrics to evaluate model performance

To evaluate the performance of our models, we randomly and independently divided the data set (*n* = 270) into two subsets: training (80%, *n* = 216) and validation (20%, *n* = 54). Loss functions (Table S3) were recorded during the execution of all three models as a measure of the difference between the predicted value and the ground truth.^49^ Accuracy curves were also acquired during runtime to compare the percentage of correct predictions achieved by each classifier.^50^ Both variables were plotted against the number of epochs to identify issues such as overfitting and underfitting.

After the execution of these models, ROC curves were generated by plotting the true positive against the false positive rate for different amounts of epochs to determine the optimal length of training.^51^, ^52^ The AUC was used as an additional metric to assess the separability, or ability to distinguish between stable and vulnerable signals, of each model. Once the optimal epoch has been selected, each classification instance from that length of training was categorized as a true positive (TP), false positive (FP), false negative (FN), or true negative (TN), depending on how the predicted condition compared against the actual condition. These values were organized into a confusion matrix, where further metrics, such as sensitivity and specificity, were calculated to assess the performance of the model more thoroughly (Methods S3). Through a holistic evaluation of all performance metrics, the three classifiers were ranked in terms of their potential for the clinical interpretation of EIS signals.

## 4 CONCLUSION

In summary, our study provides three unique contributions to existing intravascular EIS platforms: (i) a streamlined EIT reconstruction algorithm, (ii) a set of classification models to assess the metabolic vulnerability of an atherosclerotic lesion based on impedimetric data, and (iii) ex vivo testing of human carotid and coronary arteries. The streamlined EIT reconstruction algorithm was achieved by the development of a one‐step program, which processes the solution to the EIDORS forward problem and renders a conductivity map, demonstrating the radial distribution of oxLDL‐rich plaque within the arterial cross section. All reconstructions in this study were validated by immunohistological data, adding to the reliability of EIS as a method for plaque characterization. Next, we designed our classifiers from well‐established convolutional neural networks, such as ResNet and DenseNet. Despite convolutional neural networks having been traditionally used for image classification problems, we have demonstrated that they can be generalized to the data structure of an impedance spectra. We were able to train our models to predict the metabolic vulnerability of atherosclerotic lesions, with structural information from histology serving as the ground truth. Lastly, we were able to show similarities in impedimetric properties between the atherosclerotic components of pigs and that of ex vivo human arteries, whereas previous studies have largely been limited to animal models.^24^, ^25^, ^26^, ^27^ Thus, our study adds to the translational potential of intravascular EIS, paving the way towards its clinical application to predict plaque rupture in patients, in real time.

## AUTHOR CONTRIBUTIONS

**Justin Chen:** Conceptualization (lead); investigation (lead); writing – original draft (lead). **Shaolei Wang:** Data curation (equal); methodology (equal). **Kaidong Wang:** Methodology (equal). **Parinaz Abiri:** Investigation (equal); methodology (equal). **Zi‐Yu Huang:** Investigation (equal); visualization (equal). **Junyi Yin:** Visualization (lead). **Alejandro M Jabalera:** Formal analysis (equal); writing – review and editing (equal). **Brian Arianpour:** Validation (equal); writing – review and editing (equal). **Mehrdad Roustaei:** Visualization (equal). **Enbo Zhu:** Visualization (supporting). **Peng Zhao:** Visualization (supporting). **Susana Cavallero:** Resources (lead). **Sandra Duarte‐Vogel:** Investigation (equal). **Elena Stark:** Resources (equal). **Yuan Luo:** Investigation (equal). **Peyman Benharash:** Resources (equal). **Yu‐Chong Tai:** Supervision (equal). **Qingyu Cui:** Supervision (equal). **Tzung Hsiai:** Conceptualization (equal); funding acquisition (lead); supervision (lead); writing – review and editing (lead).

## CONFLICT OF INTEREST STATEMENT

The authors declare no conflicts of interest.

### PEER REVIEW

The peer review history for this article is available at https://www.webofscience.com/api/gateway/wos/peer‐review/10.1002/btm2.10616.

## Supporting information


**Data S1.** Supporting Information

## Data Availability

The data that support the findings of this study are available from the corresponding author upon reasonable request.
