# Supplementary material for: Machine learning‐directed electrical impedance tomography to predict metabolically vulnerable plaques
Source: Bioeng Transl Med. 2023 Oct 20;9(1):e10616. doi: 10.1002/btm2.10616 (PMC10771559; doi:10.1002/btm2.10616)
Supplement: Supplementary file 1 — Data S1. Supporting Information [file BTM2-9-e10616-s001.docx]

**Supplementary Materials for**

**Machine Learning-Directed Electrical Impedance Tomography to Predict Metabolically Vulnerable Plaques**

Justin Chen^1^, Shaolei Wang^1^, Kaidong Wang^2^, Parinaz Abiri^1,2^, Zi-Yu Huang^3^, Junyi Yin^1^, Alejandro M. Jabalera^1^, Brian Arianpour^1^, Mehrdad Roustaei^1^, Enbo Zhu^2^, Peng Zhao^2^, Susana Cavallero^2,4^, Sandra Duarte-Vogel^5^, Elena Stark^6^, Yuan Luo^3^, Peyman Benharash^7^, Yu-Chong Tai^3^, Qingyu Cui^2^, Tzung K. Hsiai^1,2,3,4*^

| ^1^ | Department of Bioengineering, Henry Samueli School of Engineering, University of California, Los Angeles, Los Angeles, California, 90095, USA |
| --- | --- |
| ^2^ | Division of Cardiology, Department of Medicine, David Geffen School of Medicine, University of California, Los Angeles, Los Angeles, California, 90095, USA |
| ^3^ | Department of Medical Engineering, California Institute of Technology, Pasadena, California, 91125, USA |
| ^4^ | Division of Cardiology, Department of Medicine, Greater Los Angeles VA Healthcare System, Los Angeles, California, 90073, USA |
| ^5^ | Division of Laboratory Animal Medicine, David Geffen School of Medicine, University of California, Los Angeles, Los Angeles, California, 90095, USA |
| ^6^ | Division of Anatomy, Department of Pathology and Laboratory Medicine, David Geffen School of Medicine, University of California, Los Angeles, Los Angeles, California, 90095, USA |
| ^7^ | Division of Cardiothoracic Surgery, Department of Surgery, David Geffen School of Medicine, University of California, Los Angeles, Los Angeles, California, 90095, USA |

* Corresponding author. Email: thsiai@mednet.ucla.edu

This file contains:

- Methods S1 to S3
- Figures S1 to S9
- Tables S1 to S3

**Methods S1.** Three-dimensional EIT reconstruction algorithm.

To determine the initial conductivity for the EIT reconstruction algorithm, the equation set (1) is computed below.

|  | $\frac{\text{1}}{\text{Z}_{\text{1, 3 measured}}}\text{ = }\frac{\text{1}}{\text{Z}_{\text{13}}}\text{ + }\frac{\text{1}}{\text{Z}_{\text{15}}\text{ + }\text{Z}_{\text{35}}}\text{ + }\frac{\text{2}}{\text{Z}_{\text{14}}\text{ + }\text{Z}_{\text{23}}}$  $\frac{\text{1}}{\text{Z}_{\text{3, 5 measured}}}\text{ = }\frac{\text{1}}{\text{Z}_{\text{35}}}\text{ + }\frac{\text{1}}{\text{Z}_{\text{13}}\text{ + }\text{Z}_{\text{15}}}\text{ + }\frac{\text{2}}{\text{Z}_{\text{36}}\text{ + }\text{Z}_{\text{45}}}$  $\frac{\text{1}}{\text{Z}_{\text{1, 5 measured}}}\text{ = }\frac{\text{1}}{\text{Z}_{\text{15}}}\text{ + }\frac{\text{1}}{\text{Z}_{\text{13}}\text{ + }\text{Z}_{\text{35}}}\text{ + }\frac{\text{2}}{\text{Z}_{\text{16}}\text{ + }\text{Z}_{\text{25}}}$  $\frac{\text{1}}{\text{Z}_{\text{2, 4 measured}}}\text{ = }\frac{\text{1}}{\text{Z}_{\text{24}}}\text{ + }\frac{\text{1}}{\text{Z}_{\text{26}}\text{ + }\text{Z}_{\text{46}}}\text{ + }\frac{\text{2}}{\text{Z}_{\text{23}}\text{ + }\text{Z}_{\text{14}}}$  $\frac{\text{1}}{\text{Z}_{\text{4, 6 measured}}}\text{ = }\frac{\text{1}}{\text{Z}_{\text{46}}}\text{ + }\frac{\text{1}}{\text{Z}_{\text{24}}\text{ + }\text{Z}_{\text{26}}}\text{ + }\frac{\text{2}}{\text{Z}_{\text{45}}\text{ + }\text{Z}_{\text{36}}}$  $\frac{\text{1}}{\text{Z}_{\text{2, 6 measured}}}\text{ = }\frac{\text{1}}{\text{Z}_{\text{26}}}\text{ + }\frac{\text{1}}{\text{Z}_{\text{24}}\text{ + }\text{Z}_{\text{46}}}\text{ + }\frac{\text{2}}{\text{Z}_{\text{25}}\text{ + }\text{Z}_{\text{16}}}$  $\frac{\text{1}}{\text{Z}_{\text{1, 4 measured}}}\text{ = }\frac{\text{1}}{\text{Z}_{\text{14}}}\text{ + }\frac{\text{1}}{\text{Z}_{\text{12}}\text{ + }\text{Z}_{\text{24}}}\text{ + }\frac{\text{1}}{\text{Z}_{\text{13}}\text{ + }\text{Z}_{\text{34}}}$  $\frac{\text{1}}{\text{Z}_{\text{2, 3 measured}}}\text{ = }\frac{\text{1}}{\text{Z}_{\text{23}}}\text{ + }\frac{\text{1}}{\text{Z}_{\text{12}}\text{ + }\text{Z}_{\text{13}}}\text{ + }\frac{\text{1}}{\text{Z}_{\text{24}}\text{ + }\text{Z}_{\text{34}}}$  $\frac{\text{1}}{\text{Z}_{\text{1, 6 measured}}}\text{ = }\frac{\text{1}}{\text{Z}_{\text{16}}}\text{ + }\frac{\text{1}}{\text{Z}_{\text{15}}\text{ + }\text{Z}_{\text{56}}}\text{ + }\frac{\text{1}}{\text{Z}_{\text{12}}\text{ + }\text{Z}_{\text{26}}}$  $\frac{\text{1}}{\text{Z}_{\text{2, 5 measured}}}\text{ = }\frac{\text{1}}{\text{Z}_{\text{25}}}\text{ + }\frac{\text{1}}{\text{Z}_{\text{26}}\text{ + }\text{Z}_{\text{56}}}\text{ + }\frac{\text{1}}{\text{Z}_{\text{12}}\text{ + }\text{Z}_{\text{15}}}$  $\frac{\text{1}}{\text{Z}_{\text{3, 6 measured}}}\text{ = }\frac{\text{1}}{\text{Z}_{\text{36}}}\text{ + }\frac{\text{1}}{\text{Z}_{\text{34}}\text{ + }\text{Z}_{\text{46}}}\text{ + }\frac{\text{1}}{\text{Z}_{\text{35}}\text{ + }\text{Z}_{\text{56}}}$  $\frac{\text{1}}{\text{Z}_{\text{4, 5 measured}}}\text{ = }\frac{\text{1}}{\text{Z}_{\text{45}}}\text{ + }\frac{\text{1}}{\text{Z}_{\text{34}}\text{ + }\text{Z}_{\text{35}}}\text{ + }\frac{\text{1}}{\text{Z}_{\text{46}}\text{ + }\text{Z}_{\text{56}}}$  $\frac{\text{1}}{\text{Z}_{\text{1, 2 measured}}}\text{ = }\frac{\text{1}}{\text{Z}_{\text{12}}}\text{ + }\frac{\text{2}}{\text{Z}_{\text{25}}\text{ + }\text{Z}_{\text{15}}}\text{ + }\frac{\text{2}}{\text{Z}_{\text{14}}\text{ + }\text{Z}_{\text{23}}}$  $\frac{\text{1}}{\text{Z}_{\text{3, 4 measured}}}\text{ = }\frac{\text{1}}{\text{Z}_{\text{34}}}\text{ + }\frac{\text{2}}{\text{Z}_{\text{23}}\text{ + }\text{Z}_{\text{14}}}\text{ + }\frac{\text{2}}{\text{Z}_{\text{36}}\text{ + }\text{Z}_{\text{45}}}$  $\frac{\text{1}}{\text{Z}_{\text{5, 6 measured}}}\text{ = }\frac{\text{1}}{\text{Z}_{\text{56}}}\text{ + }\frac{\text{2}}{\text{Z}_{\text{25}}\text{ + }\text{Z}_{\text{16}}}\text{ + }\frac{\text{2}}{\text{Z}_{\text{36}}\text{ + }\text{Z}_{\text{45}}}$ | (1) |
| --- | --- | --- |

This equation set produces fifteen different conductivities, which are assigned to the finite element model based on the schematic shown in **Figure S10**. This serves as the initial condition to the forward EIDORS algorithm used to compute the final conductivities.

**Methods S2.** Calculation of skewness.

To calculate the skewness ($\mũ$_3_) of our dataset, consisting of impedimetric measurements (n = 270) evaluated at the critical frequency of 50 kHz, we apply equation (1), where $X_{i}$ represents a particular sample from the dataset, $\bar{X}$ represents the mean of the distribution, and $\sigma$ is the standard deviation.

|  | $\tilde{\mu}_{3}=\frac{\sum_{i}^{N} \left( X_{i}-\bar{X} \right)^{3}}{\left( n-1 \right)\sigma^{3}}$ | (1) |
| --- | --- | --- |

A skewness of 0 indicates that the dataset is normally distributed, and a skewness greater than +0.5 or less than -0.5 signifies that the dataset is positively and negatively skewed, respectively.

**Method S3.** Calculation of performance metrics.

After each of the three models were trained on the testing dataset (n = 216), their performances were validated using a separate dataset (n = 54) and recorded in a confusion matrix. Depending on how the predicted condition compared against the actual condition, each instance was categorized into one of four cases: true positive (TP), false positive (FP), false negative (FN), and true negative (TN).

|  | | **Actual Condition** | |
| --- | --- | --- | --- |
|  |  | Vulnerable | Stable |
| **Predicted Condition** | Vulnerable | TP | FP |
|  | Stable | FN | TN |

From the values of the confusion matrix, several performance metrics were calculated using the methods shown below.

1. Accuracy

|  | $\text{Accuracy = }\frac{\text{TP}\text{ }\text{+}\text{ }\text{TN}}{\text{TP}\text{ }\text{+}\text{ }\text{FP}\text{ }\text{+}\text{ }\text{FN}\text{ }\text{+}\text{ }\text{TN}}$ | (1) |
| --- | --- | --- |

The accuracy of a model is a parameter that describes how often the classifier makes correct predictions. It is calculated by dividing the number of correctly classified instances over the total number of instances.

1. Misclassification Rate

|  | $\text{Misclassification Rate = }\frac{\text{FP}\text{ }\text{+}\text{ }\text{FN}}{\text{TP}\text{ }\text{+}\text{ }\text{FP}\text{ }\text{+}\text{ }\text{FN}\text{ }\text{+}\text{ }\text{TN}}$ | (2) |
| --- | --- | --- |

The misclassification rate of a model is a parameter that describes how often the classifier makes incorrect predictions. It is calculated by dividing the number of incorrectly classified instances over the total number of instances.

1. Sensitivity

|  | $\text{Sensitivity = }\frac{\mathrm{TP}}{\text{TP}\text{ }\text{+}\text{ }\text{FN}}$ | (3) |
| --- | --- | --- |

The sensitivity of a model, also known as the true positive rate or recall, describes how often the model classifies an instance as positive, given that it is indeed positive. It is calculated by dividing the number of correctly classified positive cases over the total number of positive cases.

1. Specificity

|  | $\text{Specificity = }\frac{\mathrm{TN}}{\text{TN}\text{ }\text{+}\text{ }\text{FP}}$ | (4) |
| --- | --- | --- |

The sensitivity of a model, also known as the true negative rate, describes how often the model classifies an instance as negative, given that it is indeed negative. It is calculated by dividing the number of correctly classified negative cases over the total number of negative cases.

1. False Positive Rate

|  | $\text{False Positive Rate}\text{ }\text{= }\frac{\text{FP}}{\text{TN}\text{ }\text{+}\text{ }\text{FP}}$ | (5) |
| --- | --- | --- |

The false positive rate of a model describes how often the model incorrectly classifies an instance as positive, given that it is negative. It is calculated by dividing the number of incorrectly classified false positive cases over the total number of negative cases.

1. Precision

|  | $\text{Precision }\text{= }\frac{\text{T}\text{P}}{\text{T}\text{P }\text{+}\text{ }\text{FP}}$ | (6) |
| --- | --- | --- |

The precision of a model describes how often the model correctly classifies an instance, given that it is positive. It is calculated by dividing the number of true positive cases over the total number of predicted positive cases.

1. F_1_ Score

|  | $\text{F}_{\text{1}}\text{ }\text{= }\frac{\text{2}}{\text{recal}\text{l}^{\text{-1}}\text{ + precisio}\text{n}^{\text{-1}}}\text{=}\frac{\text{2TP}}{\text{2TP + FP + FN}}$ | (7) |
| --- | --- | --- |

The F_1_ score is a harmonic mean of the recall and precision of the model.

**
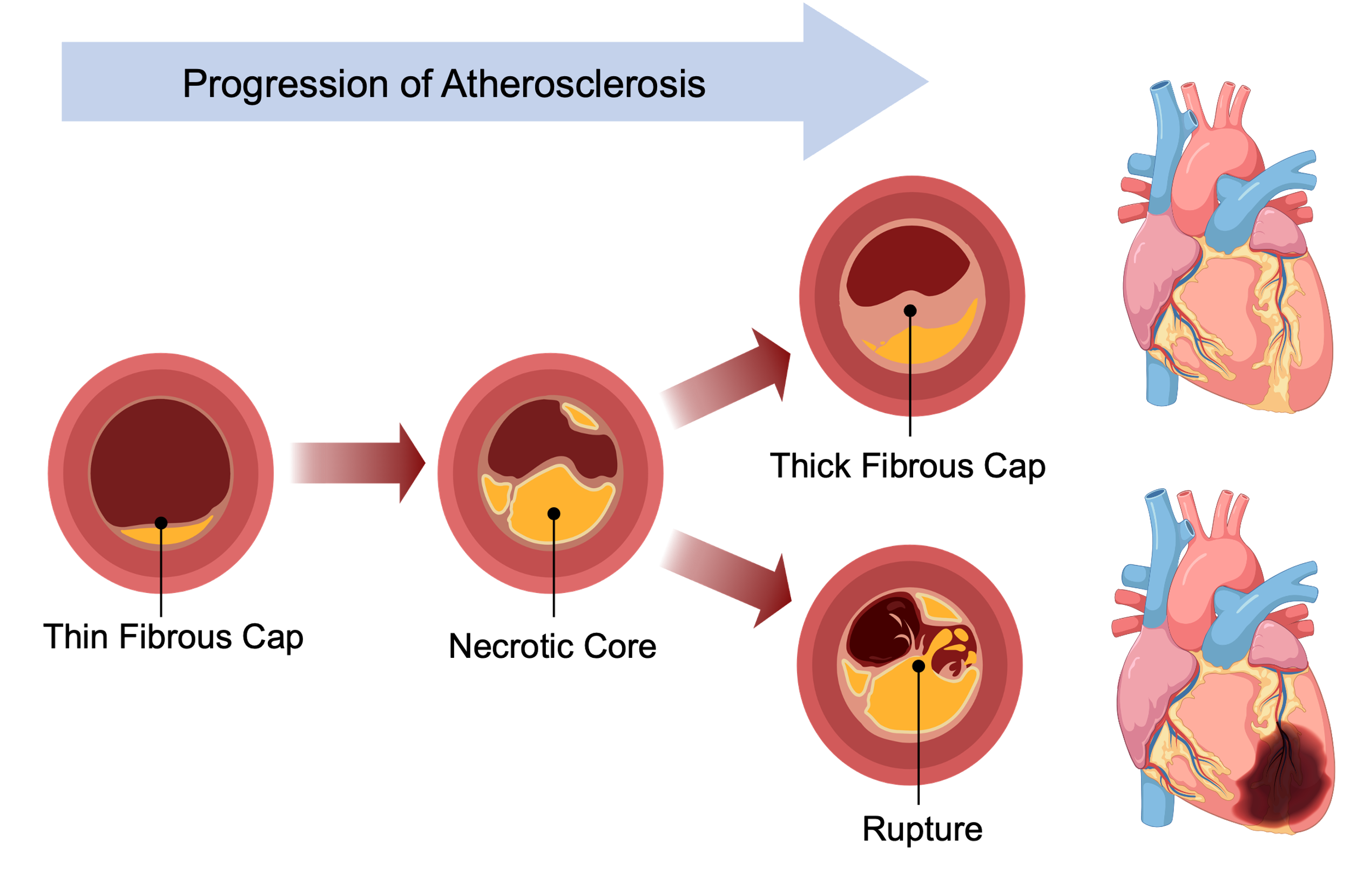
**

**Figure S1. Progression of Atherosclerosis.** Atherosclerosis is caused by an accumulation of cholesterol, fats, and other reactive substances in the endothelial layer of blood vessels. The inner layer of plaque is known as the necrotic core, which largely consists of lipids (oxLDL). Conversely, the outer layer is known as the fibrous cap, which serves as a protective barrier. Upon rupture, the contents of the necrotic core leaks into the bloodstream, causing thrombosis, and leading to a myocardial infarction or embolic stroke.

**
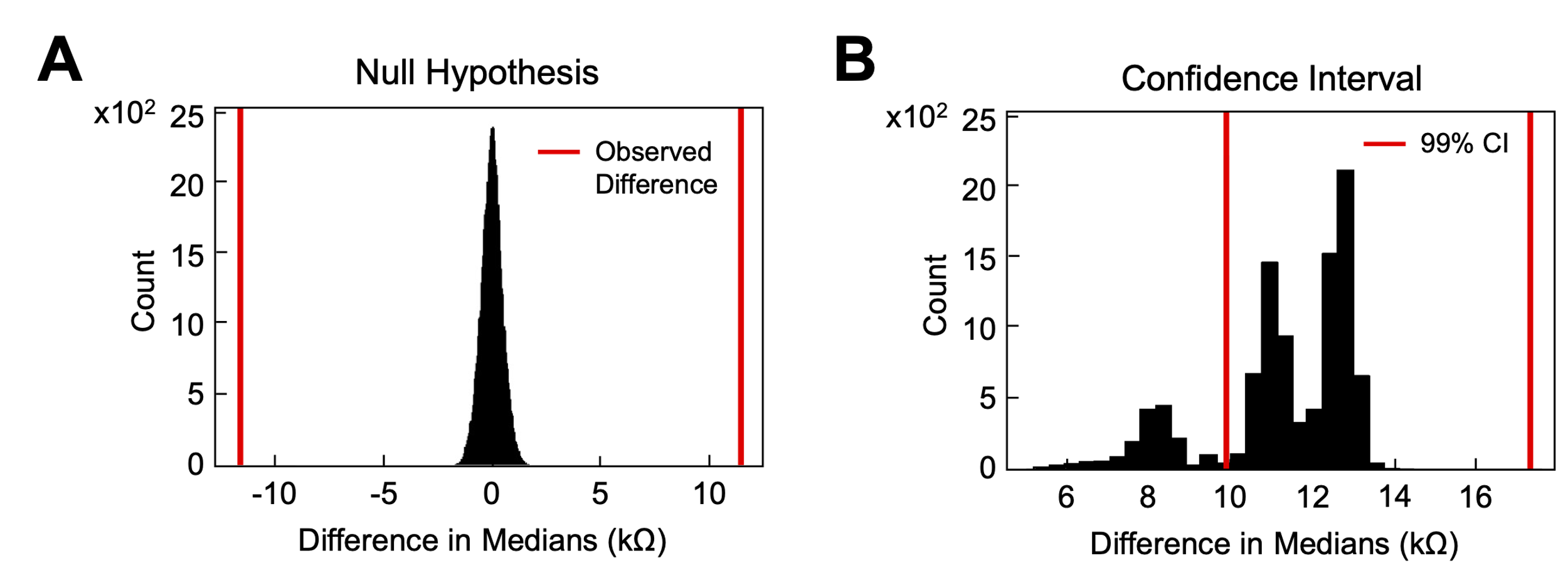
**

**Figure S2. Null hypothesis significance testing of stable and vulnerable EIS data.** (**A**) The null hypothesis ($H_{0}$) proposes that there exists no significant difference between the impedance measurements of vulnerable and stable plaques. After significance testing, the $p$-value yielded < 0.001 using difference in medians. (**B**) 99% confidence interval of the difference in medians between stable and vulnerable impedimetric data.

**
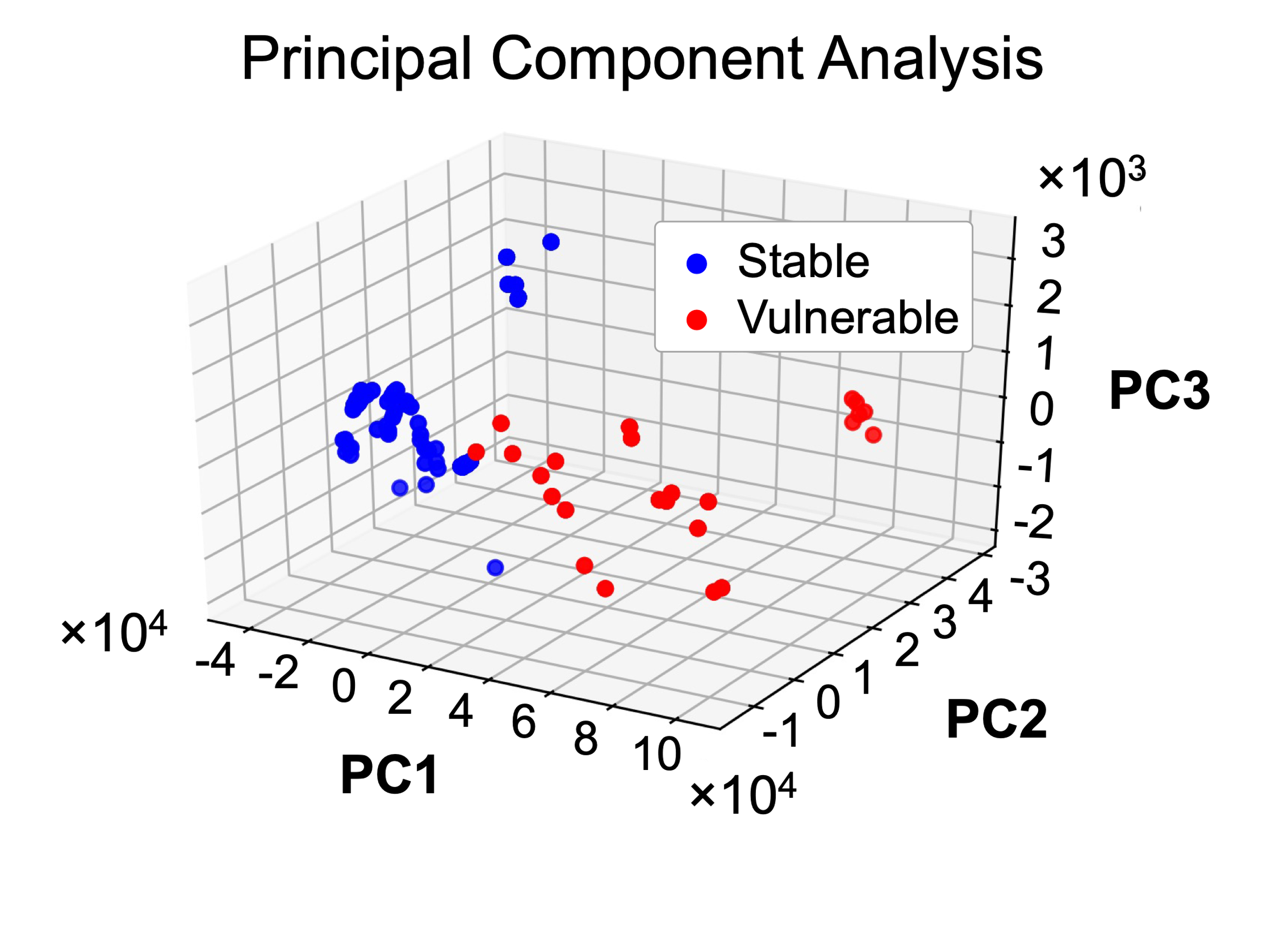
**

**Figure S3. PCA plot.** Measurements from the dataset were clustered into stable or vulnerable groups based on EIS features. Three principal components were used in this analysis.


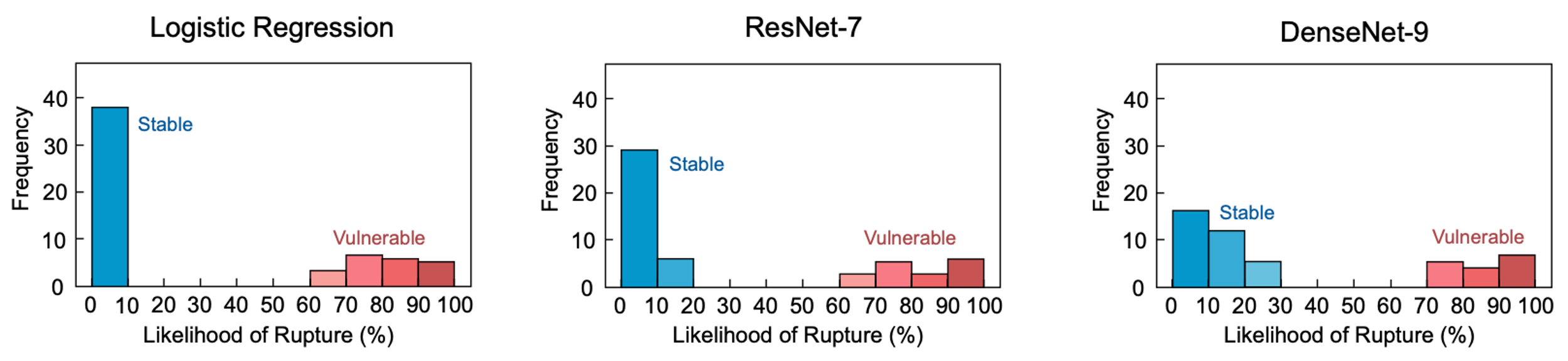


**Figure S4.** Histograms comparing the validation results of each model. The percentage likelihood of rupture is analogous to the histological plaque vulnerability index (refer to Table S2), where values above the threshold of 50 are considered vulnerable.


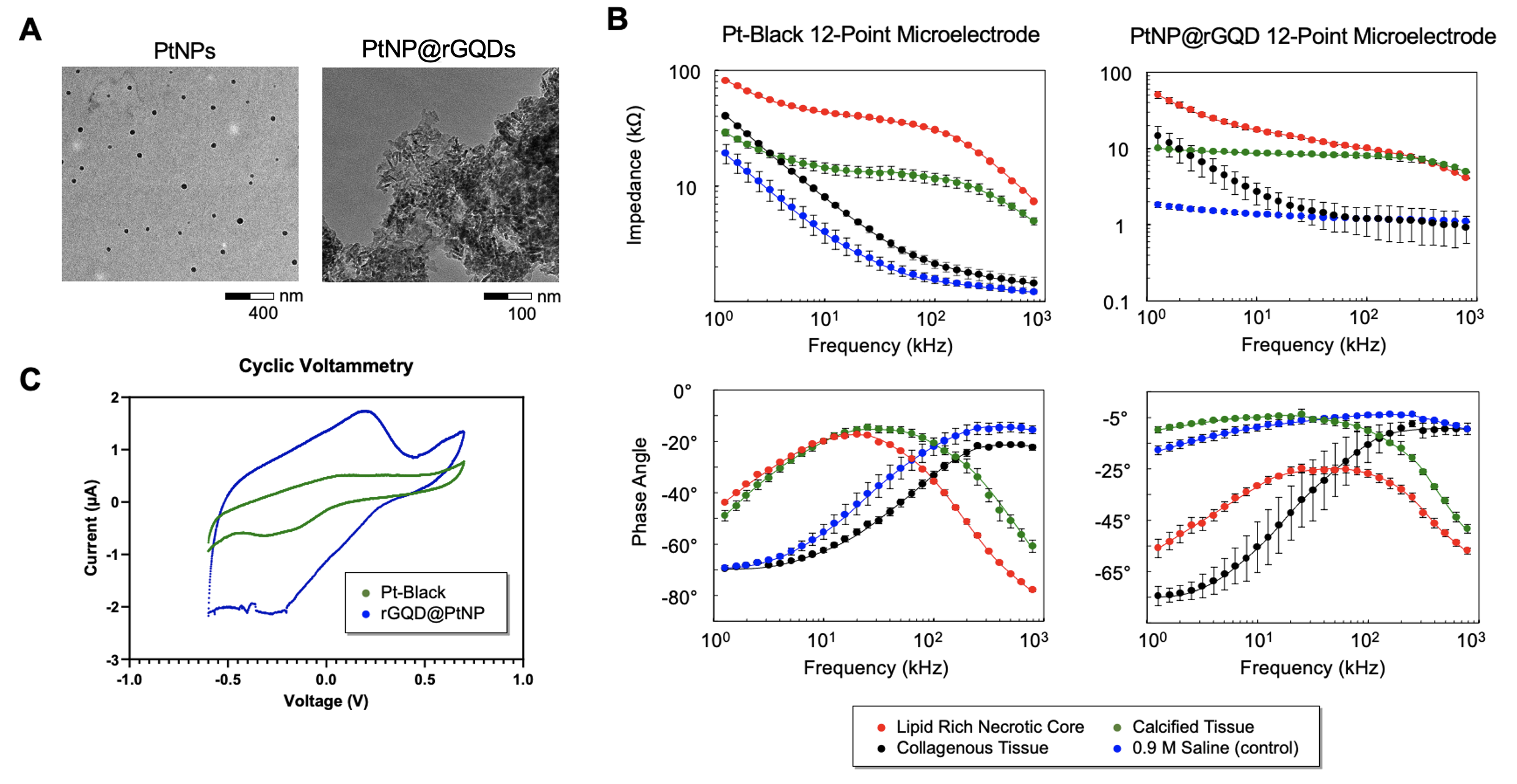


**Figure S5.** **Comparison of impedance data acquired by Pt-black-coated electrodes and composite platinum nanoparticle (PtNP@rGQD)-coated electrodes.** (**A**) TEM shows the roughness of the composite coating, suggesting that its high effective surface area can overcome the impedimetric effects of the electrochemical double layer. (**B**) Electrical impedance spectra reveals that PtNP@rGQD-coated electrodes can lower the double layer impedance at the low-frequency regime. (**C**) Cyclic voltammetry confirms the ability of PtNP@rGQD-coated electrodes to acquire impedimetric data with high sensitivity and specificity, as indicated by the larger area of its I-V curve.


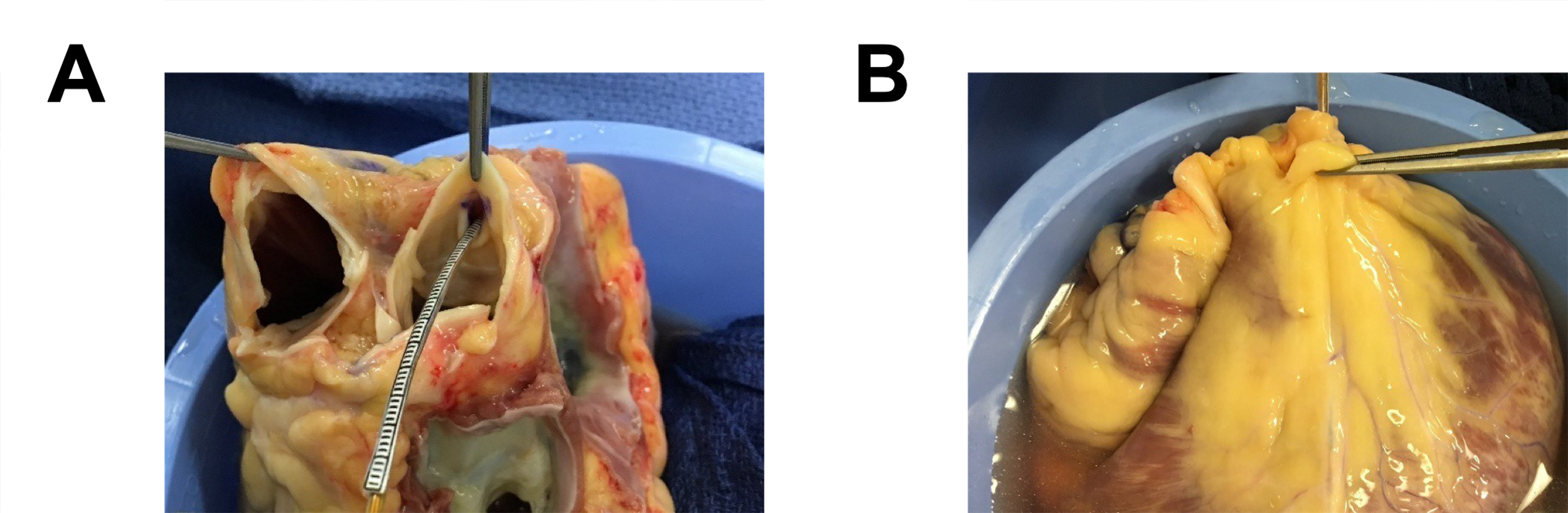


**Figure S6.** Close-up view of (**A**) the right coronary artery and (**B**) the left anterior descending artery from the explanted human heart that was used for portions of this study.


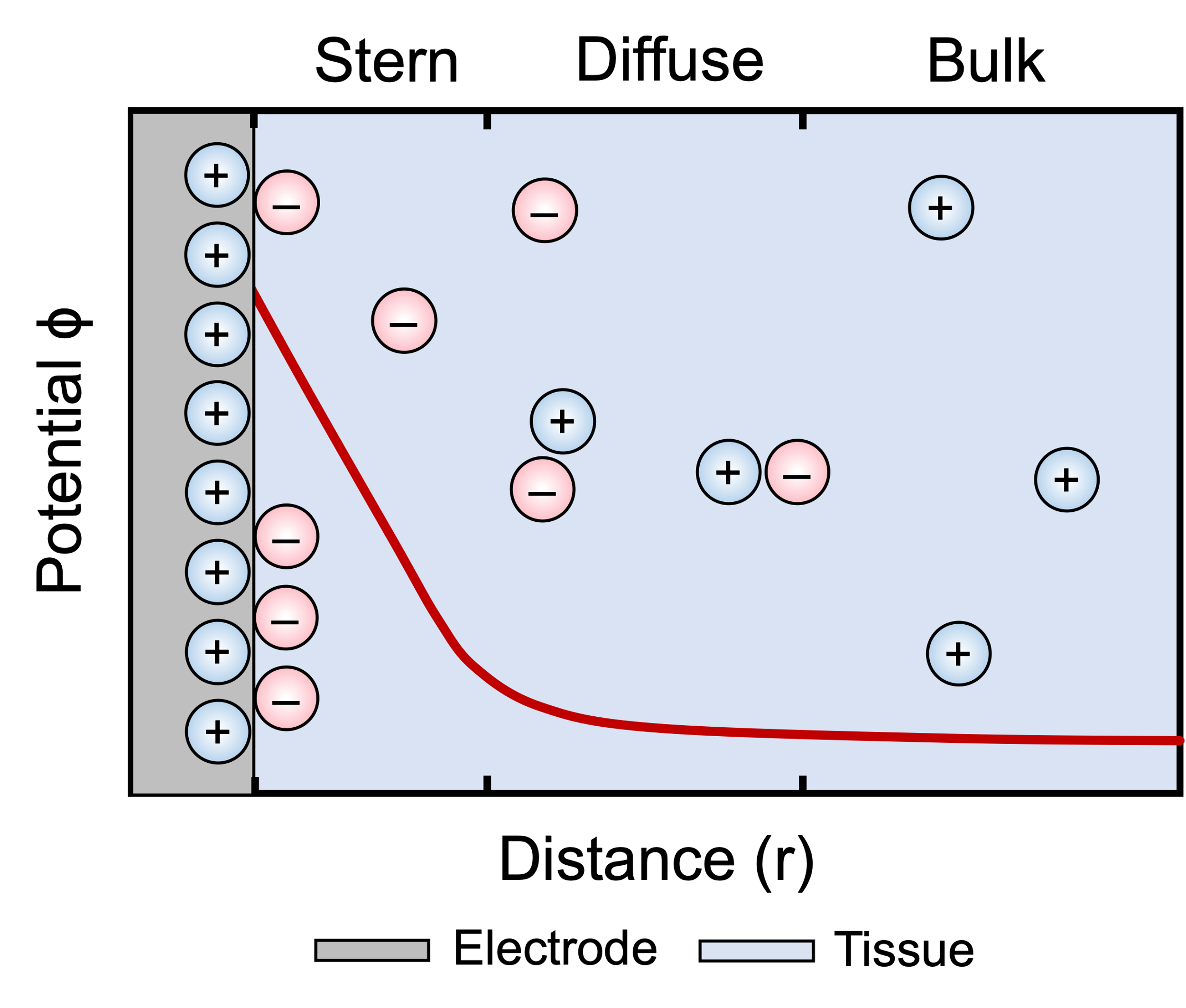


**Figure S7. Electrochemical double layer.** The electrochemical double layer refers to the behavior of ions at the electrode-tissue interface. In the Stern layer, oppositely charged ions form a compact layer and closely adhere to the surface of the electrode. The Diffuse layer, on the other hand, refers to the region where ions are free to move within the tissue. Overall, this phenomenon contributes to additional impedance during the acquisition of EIS data.


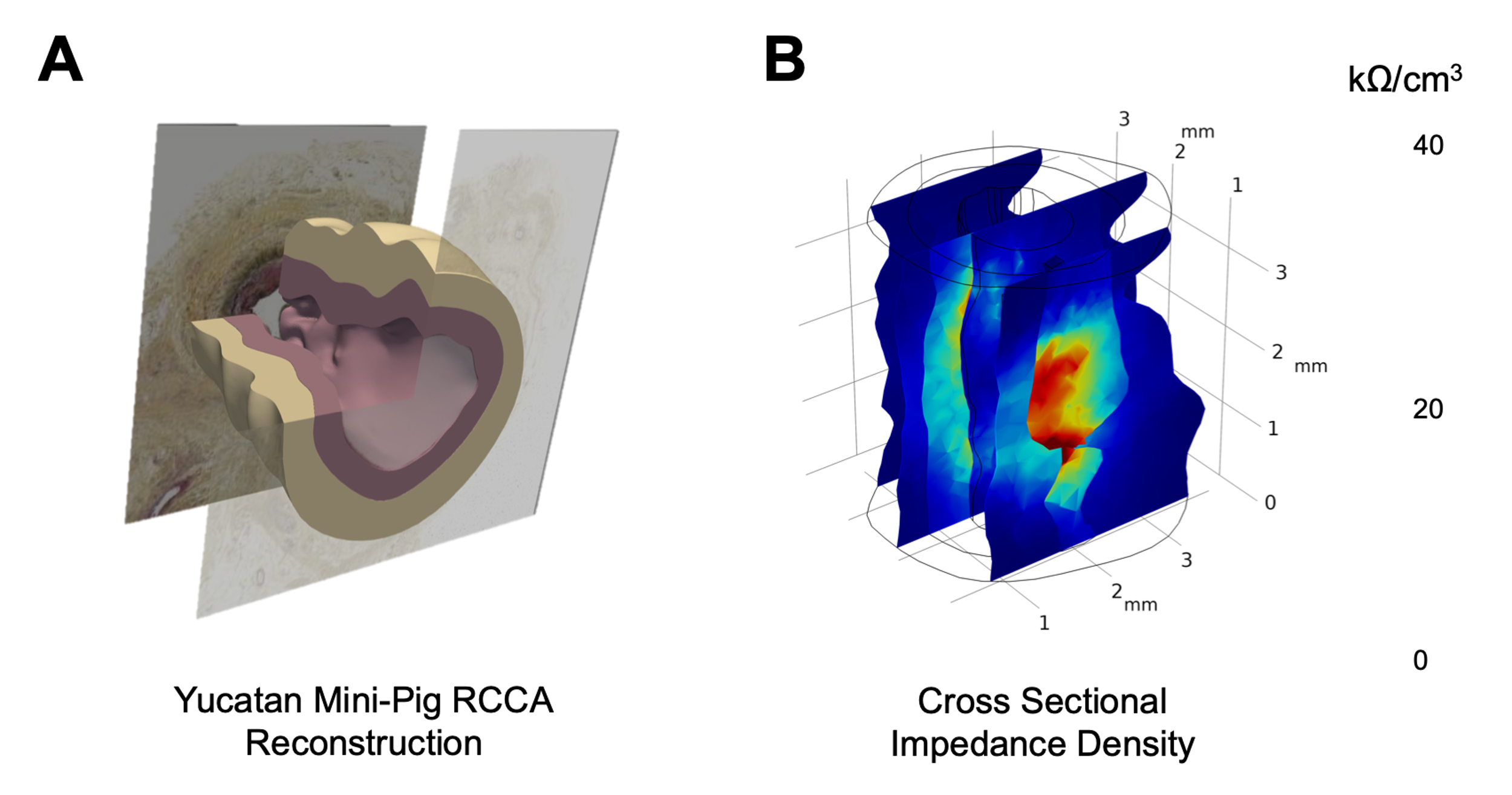


**Figure S8. Finite element analysis.** (**A**) Histology was performed on a segment of the right common carotid artery from a Yucatan mini-pig. From several two-dimensional cross sections, we generated a three-dimensional model containing structural information about the three arterial layers. (**B**) Material properties were assigned to each layer, and an impedance density map was generated with regards to the initial and boundary conditions.


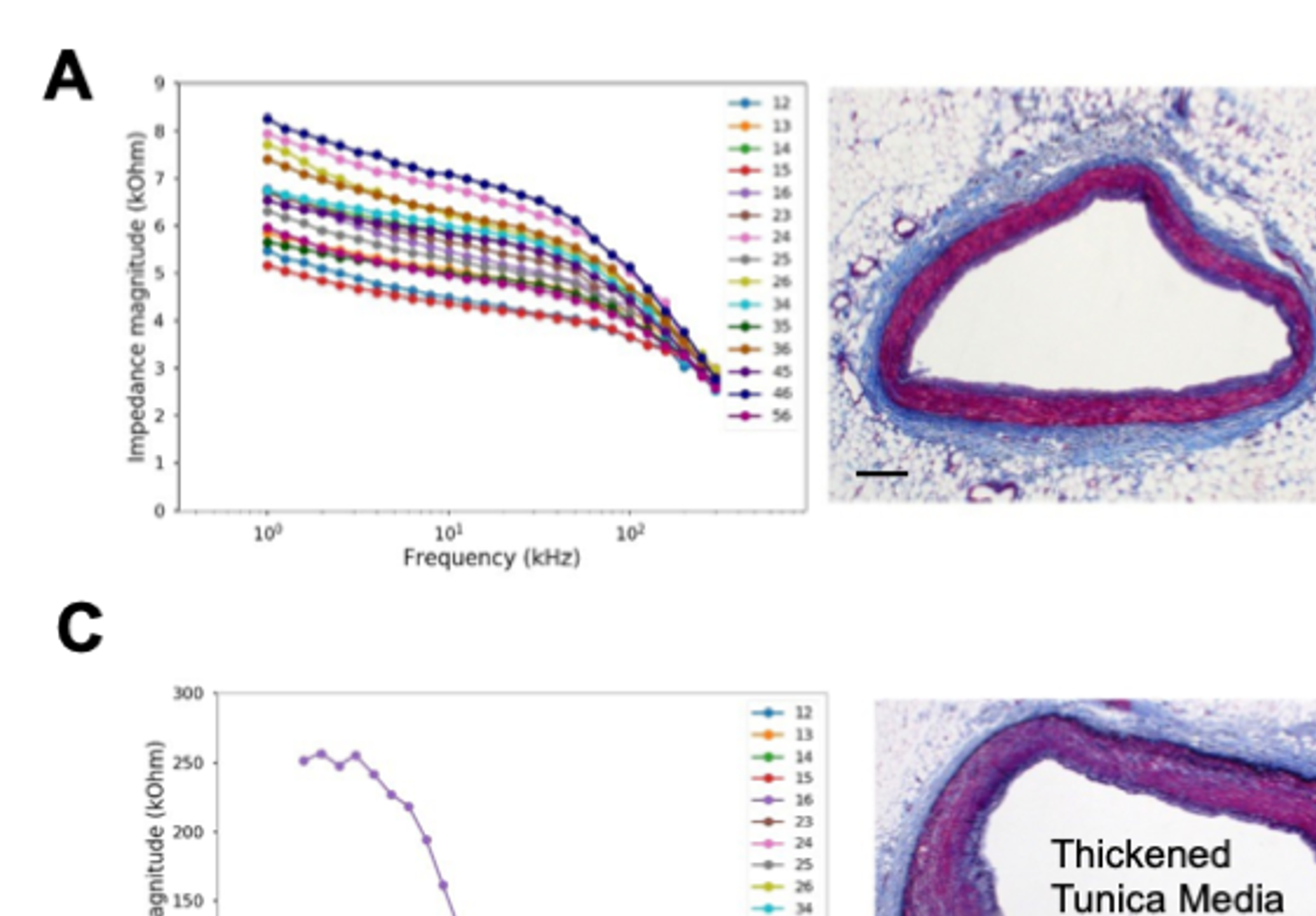


**Figure S9. Additional impedance spectra and corresponding histology from the explanted heart model.** (**A**-**D**) Right coronary artery. (**E**-**F**) Left anterior descending artery. All scale bars: 1mm.


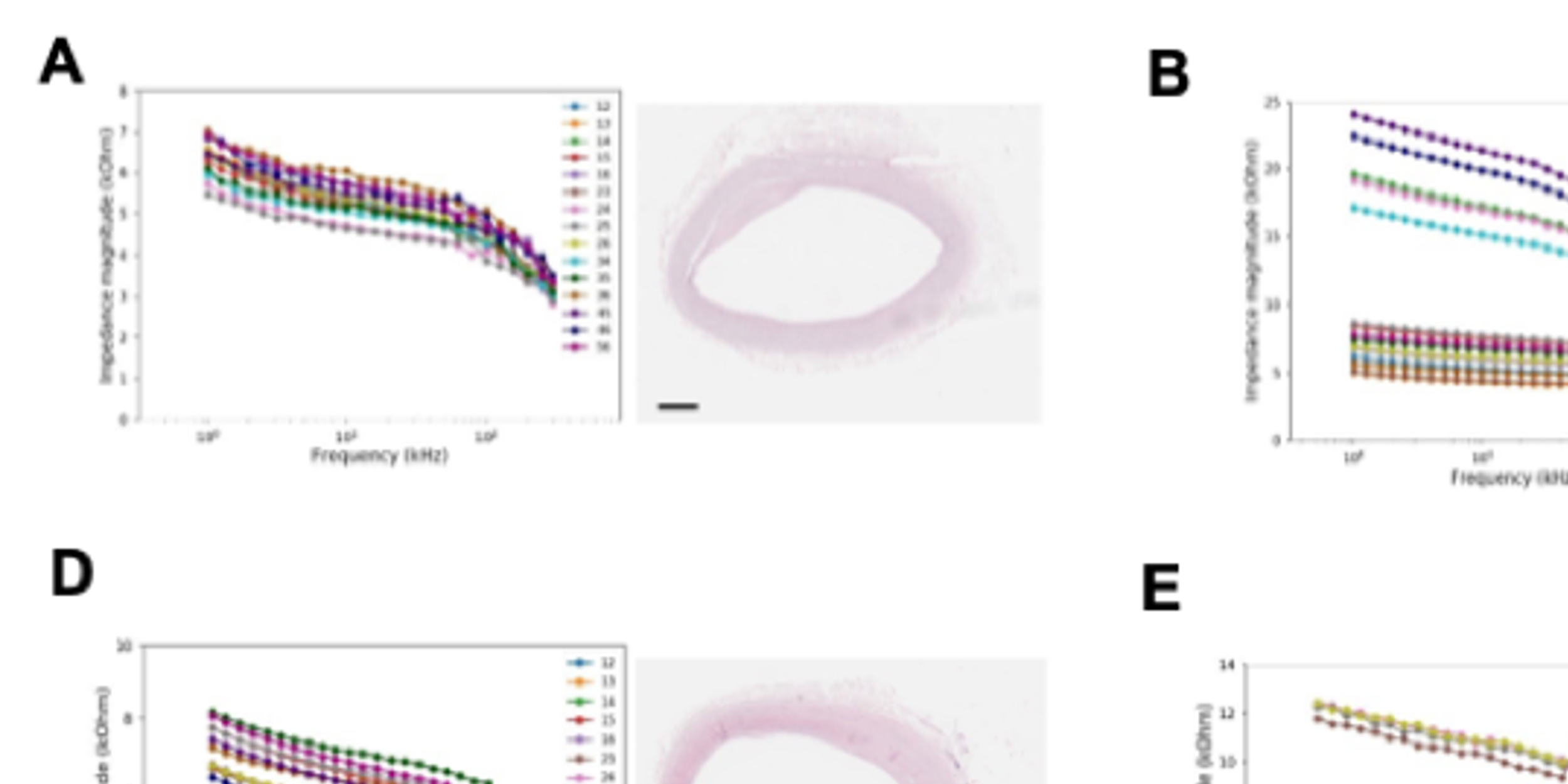


**Figure S10.** **Additional impedance spectra and corresponding histology from the human cadaver model.** (**A**-**D**) Left common carotid artery, arranged from proximal to distal. (**E**-**I**) Right common carotid artery, arranged from proximal to distal. All scale bars: 1mm.


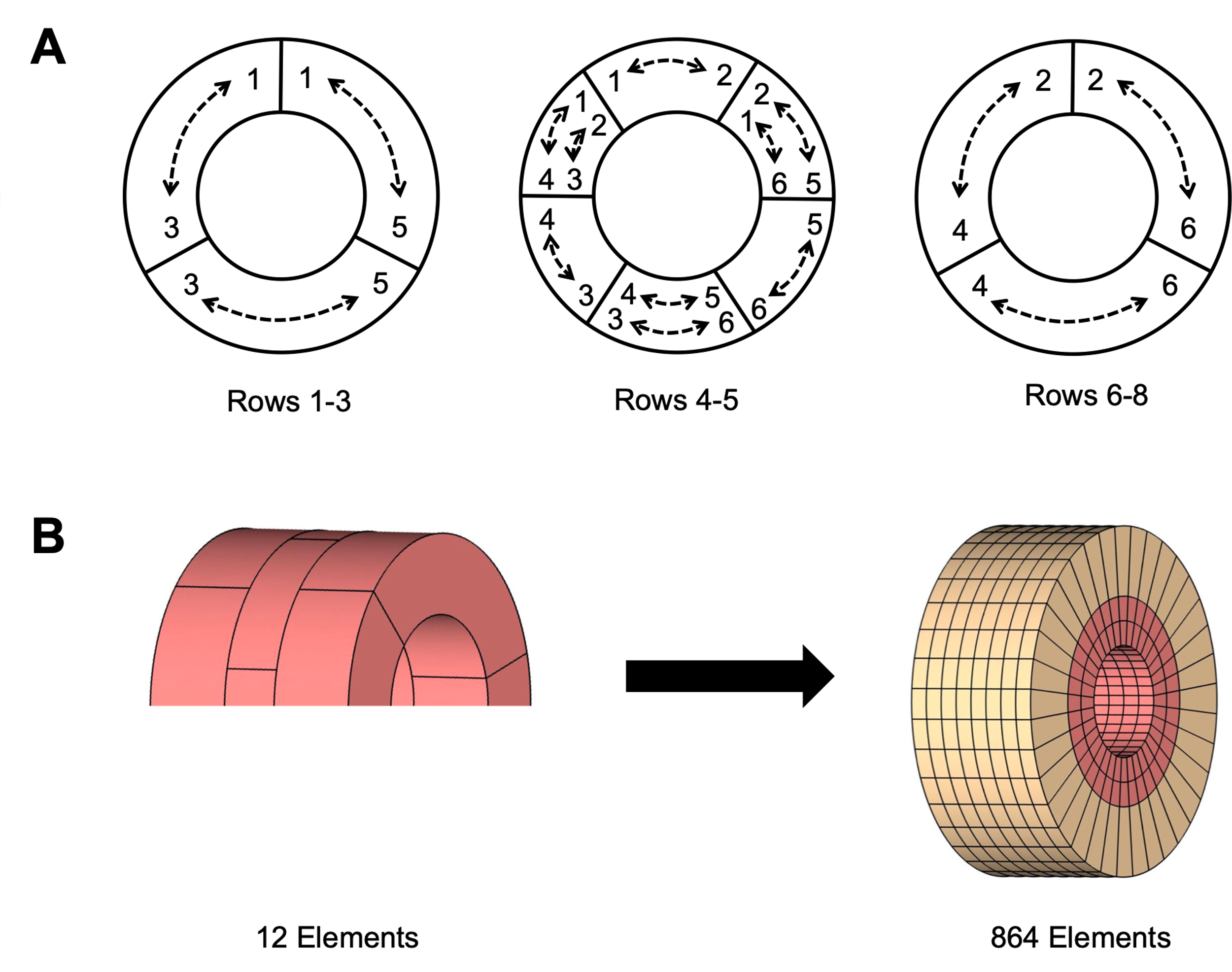


**Figure S11.** Finite element assignment. (A) A schematic of the method in which each of the fifteen computed conductivities are assigned to the FEM. Some elements on rows 4-5 contain two conductivity assignments. In such a case, their values are averaged in the initial FEM. (B) The twelve element FEM are further divided into 864 elements, in which their respective values serve as the initial conductivity matrix for the EIT reconstruction algorithm.

**Table S1.** Material properties for the three different layers of arterial wall for the computational impedance model.

|  | **Adventitia** | **Media** | **Lumen** |
| --- | --- | --- | --- |
| σ | 0.1740 | 0.3070 | 0.0417 |
| ε | 32000 | 149000 | 19300 |

* σ is expressed in units of S•m^-1^, and ε is unitless

**Table S2.** Assignment of the histological plaque vulnerability index (HPVI).

| **HPVI** | **Stability** | **Description** | **AHA Classification** |
| --- | --- | --- | --- |
| 0 | Very stable | Normal | Class I |
| 1 | Stable | Moderate intimal thickening;  No extracellular LDL, classification, or inflammation | Class II, III |
| 2 | Slightly unstable | Small lipid core (< 30% of plaque);  Little calcification;  Thick fibrous cap (> 200 µm);  Little inflammation | Class IV, Vb, Vc |
| 3 | Unstable | Lipid core (30-40% of plaque);  Fibrous cap (65 – 200 µm);  Hemorrhage and inflammation | Class Va |
| 4 | Very Unstable | Large lipid core (> 40% of plaque);  Thin fibrous cap (< 65 µm);  High levels of hemorrhage and inflammation;  Evidence of previous plaque rupture | Class VI |

* Information is adapted from Tang et al. (2005) and Stary et al. (1995)

**Table S3.** Loss functions for model evaluation.

| **Model** | **Loss Function** |
| --- | --- |
| Logistic Regression | Log |
| ResNet-7 | Binary Cross Entropy |
| DenseNet-9 | Binary Cross Entropy |
